# Analysis and Classification of Stride Patterns Associated with Children Development Using Gait Signal Dynamics Parameters and Ensemble Learning Algorithms

**DOI:** 10.1155/2016/9246280

**Published:** 2016-02-29

**Authors:** Meihong Wu, Lifang Liao, Xin Luo, Xiaoquan Ye, Yuchen Yao, Pinnan Chen, Lei Shi, Hui Huang, Yunfeng Wu

**Affiliations:** ^1^School of Information Science and Technology, Xiamen University, 422 Si Ming South Road, Xiamen, Fujian 361005, China; ^2^Department of Orthopedics, Zhongshan Hospital, Xiamen University, 201 Hubin South Road, Xiamen, Fujian 361004, China; ^3^Department of Rehabilitation, Zhongshan Hospital, Xiamen University, 201 Hubin South Road, Xiamen, Fujian 361004, China

## Abstract

Measuring stride variability and dynamics in children is useful for the quantitative study of gait maturation and neuromotor development in childhood and adolescence. In this paper, we computed the sample entropy (SampEn) and average stride interval (ASI) parameters to quantify the stride series of 50 gender-matched children participants in three age groups. We also normalized the SampEn and ASI values by leg length and body mass for each participant, respectively. Results show that the original and normalized SampEn values consistently decrease over the significance level of the Mann-Whitney *U* test (*p* < 0.01) in children of 3–14 years old, which indicates the stride irregularity has been significantly ameliorated with the body growth. The original and normalized ASI values are also significantly changing when comparing between any two groups of young (aged 3–5 years), middle (aged 6–8 years), and elder (aged 10–14 years) children. Such results suggest that healthy children may better modulate their gait cadence rhythm with the development of their musculoskeletal and neurological systems. In addition, the AdaBoost.M2 and Bagging algorithms were used to effectively distinguish the children's gait patterns. These ensemble learning algorithms both provided excellent gait classification results in terms of overall accuracy (≥90%), recall (≥0.8), and precision (≥0.8077).

## 1. Introduction

An infant commonly begins to crawl after 9 months and then learns how to walk with voluntary postural control at about one year after birth [1, 2]. During the physical growth in adolescence, the locomotor control and postural coordination of children become mature, in correspondence with the development of the central nervous system and musculoskeletal system [3]. According to Hillman et al. [4], temporal and spatial parameters of children's gait become relatively mature until 4 years old. The study of Chester et al. [5] suggested that adult-like kinetic patterns for the hip and knee are almost achieved in children by 5 years of age, whereas the ankle joint patterns remain premature until 9 years old. Menkveld et al. [6] analyzed the temporal gait parameters of a few children subjects from 7 to 16 years of age and reported that the stride patterns are more stable, but the gait modulation function still continues to improve in adolescence.

Because human locomotion functions are regulated by the neuromotor and muscular functions, immature neurological control or inconsistent muscle contractions would result in erratic body movement behaviors with irregular rhythm [7, 8]. The immature motor control of young children causes higher degree of variability in stride time (the duration from initial contact of one foot to the succeeding contact of the same foot) [4], such that the stride series would present large fluctuations or dynamic complexity. Recent studies [9, 10] emphasized how to measure the gait unsteadiness and subtle fluctuations in the course of motor skill development. Hausdorff et al. [9] used the coefficient of variation parameter and fractal analysis tools to compute the fluctuation magnitude and fractal properties of stride series of young children. Their results suggested that the stride variability significantly decreases and also exhibits long-range fractal correlations in adolescents [9].

Recently, signal irregularity analysis of physiological systems based on entropy and novel statistical measures has received extensive attractions in the research community [11, 12]. Huang et al. [13] and Wei et al. [14] measured the sample entropy (SampEn) parameter of electroencephalogram (EEG) intrinsic mode functions decomposed by the multivariate empirical mode decomposition and applied an artificial neural network trained by the back-propagation algorithm to detect the particular signal patterns related to anesthesia. Sharma et al. [15] extracted the Shannon entropy, Renyi entropy, approximate entropy, and SampEn features from the EEG signal components derived from the empirical mode decomposition algorithm and then employed the least-squares support vector machine to discriminate the focal EEG signals. In our previous studies [16, 17], we applied the nonparametric statistical methods to establish the probability density models of stride series for the adolescents at different ages.

As reported by Shumway-Cook and Woollacott [18], stride dynamics analysis may provide important indices related to the development of neuromuscular control in children. Analysis of gait patterns of young children can assist physiologists to better understand the course of gait maturation. Further quantitative studies require more advanced computational and mathematical tools to characterize the progress of gait development. The motivation of our study is to compute the SampEn and average stride interval (ASI) features to quantify the changes of gait dynamics in the stride time series of children associated with the adolescent development. The AdaBoost.M2 and Bagging ensemble learning algorithms were used to effectively perform the gait pattern classifications for the children participants in different age groups.

## 2. Material and Methods

### 2.1. Gait Data Description

The gait data set was obtained from a PhysioNet database provided by Hausdorff et al. [9], for public research of gait maturation. A total of 50 healthy children (equal number of boys and girls) aged from 3 to 14 years were recruited from the local community in Boston, MA, USA, to participate in the gait data acquisition experiments [9]. None of these children was prematurely born or suffering from any of musculoskeletal, neurological, or cardiovascular disease. In order to investigate the gait development of children with aging, the children participants were categorized into three age groups: young children of 3–5 years old (14 subjects: 6 boys and 8 girls), middle children of 6–8 years old (21 subjects: 10 boys and 11 girls), and elder children of 10–14 years old (15 subjects: 9 boys and 6 girls). Statistics of body mass and leg length of the children participants are listed in Table 1. The children's parents provided their informed and written consent letters as approved by Harvard Medical School and completed the questionnaire sheets to declare the medical history of their kids [9].

Each participant was asked to walk with his or her comfortable pace for 8 min, around a 400 m running track outdoors [9]. An investigator followed up each child during the gait data acquisition experiments. The contact force of the body on level ground was measured by two ultrathin pressure-sensitive sensors, which were placed in the right shoe of each child (one underneath the ball of the foot and the other underneath the heel) [19].

The voltage signals of force underneath the right foot were amplified with a portable signal acquisition board (dimensions: 5.5 × 2 × 9 cm; weight: 100 g) worn on the ankle cuff of each child. The signal data were sampled at 300 Hz and digitized by a built-in analog-to-digital converter with a resolution of 12 bits per sample. The series of gait cycle durations (the time from heel strike to heel strike of the same foot) or stride intervals (in seconds) were estimated with the algorithm proposed by Hausdorff et al. [19].

Because the gait speed and other phase parameters are often altered by the accelerating or decelerating movements when the subject starts or stops walking, it is necessary to eliminate the start-up or ending effects of walking posture in the gait data. In the present work, the data samples of the stride interval series recorded in the first 60 s and the last 5 s were removed, respectively, which was the same as implemented in the previous related studies [9, 17]. The stride outliers whose amplitude values were larger or smaller than three times standard deviations of the median of each stride interval series were detected and removed by a median filter [17, 20].

### 2.2. Gait Signal Dynamics Quantification

#### 2.2.1. Sample Entropy (SampEn)

SampEn has been widely used to measure the degree of regularity in complex physiological signals, by calculating the negative natural logarithm of the estimated conditional probability of self-similarity signal segments (epochs). A lower value of SampEn indicates more similar epochs occurring in the time series. Considering a gait rhythm time series {*x*(*l*)} of length *L*, we may define a template that contains a series of *k* consecutive signal elements as **x**_*m*_^*k*^ = [*x*(*m*), *x*(*m* + 1),…, *x*(*m* + *k* − 1)], where *k* is commonly known as the embedding dimension. The similar elements included in two templates are measured by the absolute maximum difference as

(1) $$\begin{array}{c} d\left[\;x_{m}^{k},x_{n}^{k}\;\right]=\underset{0\leq q\leq k-1}{\max}\left|\;x\left(\;m+q\;\right)-x\left(\;n+q\;\right)\;\right|. \end{array}$$

Let *B*_*m*_(*θ*) denote the total number of *n*,  *n* = 1,2,…, *L* − *k* + 1 (*n* ≠ *m*), which meets the requirement *d*[**x**_*m*_^*k*^, **x**_*n*_^*k*^] ≤ *θ*, where *θ* denotes the tolerance threshold for accepting the similar templates. The probability of the similar templates within the tolerance level *θ* is then defined as

(2) $$\begin{array}{c} B_{m}^{k}\left(\;\theta \;\right)=\frac{B_{m}\left(\theta \right)}{L-k+1}. \end{array}$$

Then, we can compute the average number of the total similar templates as

(3) $$\begin{array}{c} B^{k}\left(\;\theta \;\right)=\frac{1}{L-k+1}\overset{L-k+1}{\underset{m=1}{\sum }}B_{m}^{k}\left(\;\theta \;\right). \end{array}$$

Similarly, by increasing the embedding dimension up to *k* + 1, we may compute the corresponding probability of the similar templates, *A*_*m*_^*k*+1^(*θ*), as

(4) $$\begin{array}{c} A_{m}^{k+1}\left(\;\theta \;\right)=\frac{A_{m}\left(\theta \right)}{L-k}, \end{array}$$

where *A*_*m*_(*θ*) satisfies *d*[**x**_*m*_^*k*+1^, **x**_*n*_^*k*+1^] ≤ *θ*, for *n* = 1,2,…, *L* − *k* (*n* ≠ *m*). The average of all matching similar templates with the embedding dimension *k* + 1 is computed as

(5) $$\begin{array}{c} A^{k+1}\left(\;\theta \;\right)=\frac{1}{L-k}\overset{L-k}{\underset{m=1}{\sum }}A_{m}^{k+1}\left(\;\theta \;\right). \end{array}$$

Finally, the SampEn is defined as

(6) $$\begin{array}{c} \text{SampEn}\left(\;k,\theta ,L\;\right)=-\ln\left[\;\frac{A^{k+1}\left(\theta \right)}{B^{k}\left(\theta \right)}\;\right]. \end{array}$$

In the present study, the SampEn method was used to probe the self-similarity gait signal epochs by estimating the similar-matching templates in stride series. The length of stride series *L* = 350 is identical for every single child. The SampEn embedding dimension is set to be *k* = 2. The optimal tolerance parameter of the SampEn model, *θ* = 0.05, was derived with the lowest *p* value results of the Mann-Whitney *U* test (significance level: *p* < 0.01). Thus, the SampEn(2, 0.05, 350) model was selected to quantify the gait regularity in the children's stride series.

#### 2.2.2. Average Stride Interval (ASI)

ASI is referred to as the mean of stride interval during a period of gait monitoring [17]. In the present work, we computed the ASI value based on the probability density function (PDF) of stride interval, as a dominant gait feature to represent the average duration of a stride for each child participant. The PDF of stride interval provides a continuous probability distribution estimate for a number of stride observations. For a given stride time series {*x*(*l*)},  *l* = 1,2,…, *L*, the PDF of stride interval, $\hat{p}(g)$, can be established by using the Parzen-window method [17, 20, 21] as

(7) $$\begin{array}{c} \hat{p}\left(\;x\;\right)=\frac{1}{L}\overset{L}{\underset{l=1}{\sum }}\kappa \left[\;x-x\left(\;l\;\right)\;\right], \end{array}$$

where *κ*(·) denotes a nonnegative kernel function, which integrates to unity; that is, ∫_−*∞*_^*∞*^ *κ*(*x*)*dx* = 1.

In our study, the prevailing Gaussian kernel function was applied to estimate the PDF of stride interval; that is,

(8) $$\begin{array}{c} \kappa \left[\;x-x\left(\;l\;\right)\;\right]=\frac{1}{\sqrt{2\pi }\sigma }\exp\left\{\;\frac{-{\left[x-x\left(l\right)\right]}^{2}}{2\sigma^{2}}\;\right\}, \end{array}$$

where *σ* denotes the spread parameter of the Gaussian function. It is worth noting that the center of the Gaussian function is located at the amplitude of each stride observation *x*(*l*), and the spread parameter *σ* determines the Gaussian kernel window width [20].

The effectiveness of nonparametric PDF estimate by means of the Parzen-window method depends on the optimal choice of the spread parameter [22]. In order to select the best spread parameter, the estimated PDF was compared with the histogram of stride interval with the same resolution; that is, the discrete scale of the stride PDF is equal to the number of histogram bins. In the searching range of [0.001,0.1], with an increment step of 0.001, the spread parameter of 0.01 that matched the minimization criterion of the mean-squared error between the Parzen-window PDF and the histogram of stride interval was chosen as the optimal value [22]. Then, the ASI value can be calculated as the mean of stride interval based on the estimated Parzen-window PDF [17] as

(9) $$\begin{array}{c} \text{ASI}=\overset{\infty }{\underset{-\infty }{\int }}x\hat{p}\left(\;x\;\right)dx. \end{array}$$

We computed the ASI values for all 50 children participants and also applied the Mann-Whitney *U* test (implemented with IBM SPSS Statistics, Version 20) to study the statistical differences of ASI among three different age groups (significance level: *p* < 0.01).

### 2.3. Ensemble Learning Algorithms

With the SampEn and ASI features obtained, we may perform effective gait pattern classifications for further analysis. For two decades, multiple learner systems trained by advanced ensemble learning algorithms have received extensive attentions in the machine learning community [23–26]. Ensemble learning is also referred to as committee machine learning, which follows a so-called “divide-and-conquer” strategy [27]. An ensemble paradigm commonly divides a complex classification or regression problem into a few simple tasks with lower computational expense and then combines a group of trained component learners to provide a comprehensive solution [28]. In the present work, we used the Boosting and Bagging algorithms, two most popular ensemble learning paradigms, to distinguish the gait patterns of the children participants into three age groups.

#### 2.3.1. AdaBoost Algorithm

Boosting algorithms work by sequentially generating a number of weak learners to solve a classification or regression problem together [29]. In a typical boosting procedure, the training data for each weak learner are regenerated in order to correct the mistakes made by the previous learner. The AdaBoost algorithm is a representative boosting method that intends to accomplish the training of weak learners by reweighting or resampling the data samples [30]. Researchers have developed the family of AdaBoost algorithms with plenty of extension versions, such as AdaBoost.R [30], AdaBoost.M1 [31], and AdaBoost.M2 [32], to solve different types of regression or classification problems. In the present work, we implemented the AdaBoost.M2 ensemble method that involved a total of 50 decision trees as the base learners to implement the gait pattern classifications. The computation process of the AdaBoost.M2 algorithm for the classification of children's gait patterns is summarized as follows.

*Computation Process of the AdaBoost.M2 Algorithm*

 
*Input:*
   Gait data set: {**f**_*n*_, *t*_*n*_}_*n*=1_^*N*^,  *N* = 50 is the number of children, *t*_*n*_ ∈ {1,2, 3} is the class label;
   Weak learner model (decision tree): *h*(**f**_*n*_);
   Number of ensemble learning iteration: *I*.
 
*Initialization:*
   Initialize the gait data distribution *ℓ*_1_(**f**_*n*_) = 1/*N*.
 
*Computation Procedure:*
  (1) *for* *i* = 1,2,…, *I:*
  (1) Train a weak learner *h*_*i*_(**f**_*n*_).
  (3) Calculate the error of the *i*th classifier: *e*_*i*_ = ∑_*n*:*h*_*i*_(**f**_*n*_)≠*t*_*n*__ *ℓ*_*i*_(**f**_*n*_).
  (4) Set *α*_*i*_ = (1/2)ln⁡((1 − *e*_*i*_)/*e*_*i*_).
  (5) Update the distribution:
    (10) $$\begin{array}{c} l_{i+1}\left(\;f_{n}\;\right)=\frac{l_{i}\left(f_{n}\right)}{Z_{i}}\left\{\begin{array}{cc} \exp\left(-\alpha_{i}\right), & \text{if }h_{i}\left(f_{n}\right)=t_{i},\\ \exp\left(\alpha_{i}\right), & \text{if }h_{i}\left(f_{n}\right)\neq t_{i}, \end{array}\right. \end{array}$$
   where *Z*_*i*_ is a normalization constant that makes *ℓ*_*i*+1_(**f**_*n*_) be a probability distribution.
  (6) *end*
 
*Output:*
  (11) $$\begin{array}{c} H_{\text{AdaBoost}}\left(\;f_{n}\;\right)=\mathrm{sign}\left(\;\overset{I}{\underset{i=1}{\sum }}\alpha_{i}h_{i}\left(\;f_{n}\;\right)\;\right). \end{array}$$

#### 2.3.2. Bagging Algorithm

Bagging stands for “bootstrap aggregating” [33], which contains the procedures of bootstrap sampling of training data, and aggregation of base learners by voting for classification problem or averaging for regression problem. The Bagging algorithm is able to greatly improve the generalization capability by combining weak learners (e.g., decision trees), rather than stable learners (such as *k*-nearest neighbor classifiers, radial basis function networks, and support vector machines), which are insensitive to the adjustment of training data with a bootstrap distribution [33].

Given a data set containing *N* scatter points (gait patterns), the bootstrap sampling approach generates a new training data set of the same size, **f**_*n*_^bd^, for each weak learner by random (the Monte Carlo method) sampling from the original data set **f**_*n*_ [26]. In the bootstrap sampling process, a data point (or gait pattern) is picked with the uniform probability, 1/*N*, irrespective of whether being selected before or not. Such a bootstrap sampling mechanism may result in several data points appearing more than once, whereas some other points are replaced with these repetitions in the new training data set. When predicting a testing gait pattern, the Bagging algorithm aggregates the outputs of the weak learners by voting the class labels and then makes the most voted label as the ensemble decision [34]. Breiman [33] demonstrated that the generalization error of the Bagging ensemble would be greatly reduced in comparison with the prediction error of a single base learner. In the present study, we used the Bagging algorithm that combined 50 weak learners in the form of decision trees (the same number of learners as that of the AdaBoost.M2 algorithm for comparison purpose), to accomplish the children's gait pattern classification tasks. The detailed computation process of the Bagging algorithm is provided as follows.

*Computation Process of the Bagging Algorithm*

 
*Input:*
   Gait data set: {**f**_*n*_, *t*_*n*_}_*n*=1_^*N*^,  *N* = 50 is the number of children, *t*_*n*_ ∈ {1,2, 3} is the class label;
   Weak learner model (decision tree): *h*(**f**_*n*_);
   Number of weak learners: *I*.
 
*Computation Procedure:*
  (1) *for* *i* = 1,2,…, *I:*
  (2) Train a weak learner *h*_*i*_(**f**_*n*_^bd^) with a data set of bootstrap distribution **f**_*n*_^bd^.
  (3) Predict the class labels of the input patterns with the trained learners *h*_*i*_(**f**_*n*_; **f**_*n*_^bd^).
  (4) *end*
 
*Output:*
  (12) $$\begin{array}{c} H_{\text{Bagging}}\left(\;f_{n}\;\right)=\underset{t\in \left\{1,2,3\right\}}{\arg\max}\overset{I}{\underset{i=1}{\sum }}\left[\;h_{i}\left(\;f_{n};f_{n}^{bd}\;\right)=t\;\right]. \end{array}$$

### 2.4. Classification Performance Evaluation

With the purpose of categorizing children's gait patterns into multiple classes (three age groups), we considered the one-versus-rest strategy, which makes the classifiers train and test with the patterns of a specified class as positive cases and all other cases as negative ones. Such a classification process was alternately implemented for each class. The classification results of the AdaBoost.M2 and Bagging algorithms were then evaluated with the recall, precision, and accuracy metrics. Let TP_*t*_, FP_*t*_, and *N*_*t*_ denote the number of true positive (correct classification) cases, the number of predicted positive cases, and the total number of cases for a specified class (*t* ∈ {1,2, 3}), respectively. Recall is defined as the true positive rate or sensitivity; that is,

(13) $$\begin{array}{c} {\text{Recall}}_{t}=\frac{{\text{TP}}_{t}}{N_{t}}. \end{array}$$

Precision represents the positive predictive value, which is expressed as

(14) $$\begin{array}{c} {\text{Precision}}_{t}=\frac{{\text{TP}}_{t}}{{\text{TP}}_{t}+{\text{FP}}_{t}}. \end{array}$$

Accuracy is the percentage ratio of all correct classified cases over the total number of cases:

(15) $$\begin{array}{c} Accuracy=\frac{\sum_{t=1}^{3}{\text{TP}}_{t}}{\sum_{t=1}^{3}N_{t}}\times 100\%. \end{array}$$

## 3. Results and Discussions

**Figure 1:**
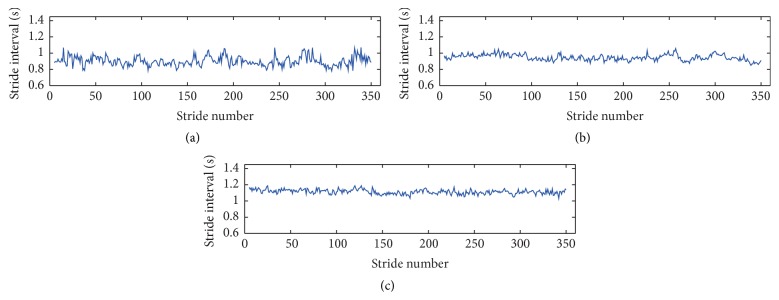
Series of stride interval of the children (a) aged 47 months, (b) aged 88 months, and (c) aged 148 months, respectively. The first strides come after the start-up walking for 60 s, and the strides during the last 5 s walking are excluded in the stride series.

Figure 1 plots the series of stride interval of three children in the corresponding age groups, respectively. The beginning strides in the first 60 s and the ending strides in the final 5 s during the gait monitoring period have been excluded in the gait series records. The outliers were also removed in the stride series by the median filter developed by Wu and Krishnan [20].

**Figure 2:**
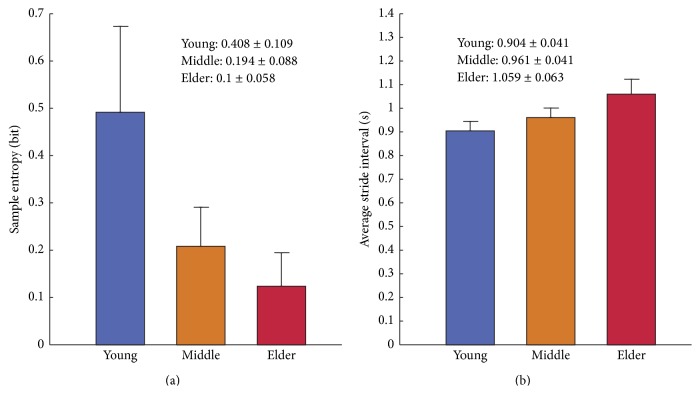
Statistics of (a) sample entropy (SampEn) and (b) average stride interval (ASI) of the children in the young (3–5 years old), middle (6–8 years old), and elder (10–14 years old) age groups, respectively.

Figure 2 shows different SampEn and ASI values in bar graphics for the children participants in three age groups. It can be observed that the SampEn values consistently decrease from 0.408 bits (young age group) to 0.194 bits (middle age group), until 0.1 bits (elder age group). However, the ASI values slightly raise from 0.904 s (young children) to 0.961 s (middle children), until 1.059 s (elder children). Reduction of the SampEn results indicates that the irregularity in the series of stride interval has been ameliorated with the body maturation in children. Increase of the ASI values suggests that the children participants are able to coordinate larger strides when they grow up.

In the present study, we also normalized the SampEn and ASI parameters by the leg length and body mass for each participant, respectively. Statistical results of the original and normalized SampEn values, along with the original and normalized ASI values, for the children in the young, middle, and elder age groups are provided in Table 2.

It is clear that the changes of the original SampEn and ASI parameters between any two age groups are over the statistical significance level of the Mann-Whitney *U* test (*p* < 0.01). The SampEn normalized by leg length significantly reduces more than a half, from 0.755 bits/m to 0.304 bits/m, when the children grow up until 8 years old. For the children aged 10–14 years, the SampEn value normalized by leg length becomes 0.129 bits/m on average, with a decrement of 0.626 bits/m versus that of the young children aged 3–5 years. The SampEn normalized by body mass also decreases from 0.023 to 0.003 bits/kg for the children aged 10–14 years. Such results indicate that the gait irregularity, parameterized with the normalized SampEn by leg length and body mass, has been greatly improved in a close relationship with the maturation of motor control and musculoskeletal development in adolescence. The gait irregularity is reduced rapidly when the children become 8 years old, and the stride variability continues to decrease in children until the age of 14 years. The ASI values normalized by leg length and body mass are consistently becoming smaller in children with aging over the significance level (*p* < 0.01). However, the original ASI value is with an increasing trend, which is different from the normalized values. Such results indicate that the musculoskeletal development and gain in weight are more remarkable than the increase of stride interval in children. Both of the SampEn and ASI results suggest that the growth of musculoskeletal and neurological systems enable the children to better modulate the gait cadence rhythm, which confirms the observations in previous related studies of Hausdorff et al. [9] and Xiang et al. [17].

**Figure 3:**
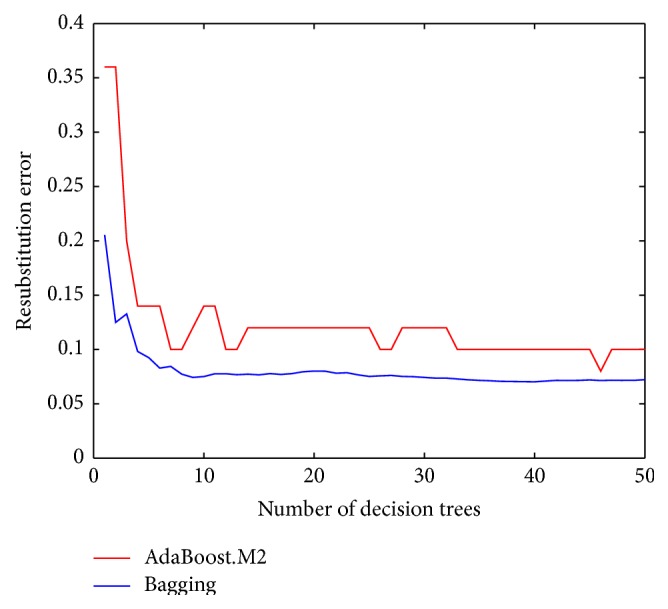
Resubstitution errors of the ensembles in relation to the increasing number of decision trees that are involved in the AdaBoost.M2 and Bagging algorithms, respectively.

The gait pattern classification results are tabulated in Table 3. Both of the AdaBoost.M2 and Bagging algorithms provided excellent overall accurate rates (AdaBoost.M2: 90%, Bagging: 92%). The Bagging algorithm correctly categorized all 14 gait patterns in the young children group, whereas the AdaBoost.M2 algorithm misclassified a child of 45 months after birth into the middle age group. Thus, the Bagging algorithm outperformed the AdaBoost.M2 algorithm with better results in terms of recall (Bagging: 0.9286 versus AdaBoost.M2: 0.8571) and precision (Bagging: 0.84 versus AdaBoost.M2: 0.8077).  Figure 3 displays the resubstitution errors produced by the AdaBoost.M2 and Bagging algorithms when generating new decision tree learners. It is worth noting that both ensemble methods can greatly reduce the output errors. The error curve of the Bagging algorithm is consistently below that of the AdaBoost.M2 algorithm, which confirms the effectiveness and superiority of the Bagging algorithm for solving the children's gait pattern classification problem.

## 4. Conclusion

Computer-aided quantification of stride dynamics and analysis of gait patterns may provide useful information on the neuromotor development in adolescence. In the present work, the SampEn and ASI parameters were computed to investigate the degree of gait regularity and the average gait cadence duration in children. The SampEn parameter can adapt to a small length of gait signal, such that it is not necessary to require the children participants to walk for a long-term gait monitoring. It is therefore very suited for the gait maturation assessment in adolescents, especially for young children who may have muscular fatigue in long-distance walking. Our results show that the SampEn and ASI values are significantly changing in adolescents aged from 3 to 14 years. The classification results demonstrated the effectiveness of the AdaBoost.M2 and Bagging ensemble algorithms in the identification of gait patterns for the children in different age groups. In the future study, we plan to recruit more gender-matched children participants in the three age groups for more accurate and unbiased statistical analysis of gait patterns during short-term and long-term walking monitoring. More temporal and computational tools [28] would be considered to analyze other stride phases, such as stance interval, swing interval, and double support time.

## Figures and Tables

**Table 1:** Statistics of body mass and leg length of the children in the young, middle, and elder age groups, respectively. Values are expressed as mean ± standard deviation.

Age groups	Body mass (kg)	Leg length (m)
Young (3–5 years old)	18.01 ± 2.98	0.55 ± 0.04
Middle (6–8 years old)	25.31 ± 4.02	0.65 ± 0.05
Elder (10–14 years old)	42.61 ± 9.21	0.79 ± 0.07

**Table 2:** Statistics of the original and normalized SampEn(2, 0.05, 350) and the original and normalized ASI values for the children in the young, middle, and elder age groups. Statistical differences between pairs of age groups are evaluated by the Mann-Whitney *U* hypothesis test (significance level *p* < 0.01). SampEn: sample entropy. ASI: average stride interval. *∗*: *U* test between the young and middle age groups; *∗∗*: *U* test between the middle and elder age groups; *∗∗∗*: *U* test between the young and elder age groups.

Entropy parameters	Statistics (mean ± standard deviation)			*p* value
	Young group	Middle group	Elder group	
	(aged 3–5 years)	(aged 6–8 years old)	(aged 10–14 years)	
SampEn (bit)	0.408 ± 0.109	0.194 ± 0.088	0.1 ± 0.058	0.001^*∗*^
				0.001^*∗∗*^
				0.001^*∗∗∗*^

Normalized SampEn by leg length (bit/m)	0.755 ± 0.229	0.304 ± 0.139	0.129 ± 0.084	0.001^*∗*^
				0.001^*∗∗*^
				0.001^*∗∗∗*^

Normalized SampEn by body mass (bit/kg)	0.023 ± 0.008	0.008 ± 0.003	0.003 ± 0.002	0.001^*∗*^
				0.001^*∗∗*^
				0.001^*∗∗∗*^

ASI (s)	0.904 ± 0.041	0.961 ± 0.041	1.059 ± 0.063	0.004^*∗*^
				0.001^*∗∗*^
				0.001^*∗∗∗*^

Normalized ASI by leg length (s/m)	1.661 ± 0.132	1.495 ± 0.122	1.35 ± 0.106	0.001^*∗*^
				0.002^*∗∗*^
				0.001^*∗∗∗*^

Normalized ASI by body mass (s/kg)	0.051 ± 0.008	0.039 ± 0.005	0.026 ± 0.004	0.001^*∗*^
				0.001^*∗∗*^
				0.001^*∗∗∗*^

**Table 3:** Gait pattern classification results obtained by the AdaBoost.M2 and Bagging ensemble methods.

Classification evaluation metrics	Ensemble methods	
	AdaBoost.M2	Bagging
Accuracy (%)	90%	92%
Recall		
Young (3–5 years old)	0.8571	0.9286
Middle (6–8 years old)	1	1
Elder (10–14 years old)	0.8	0.8
Precision		
Young (3–5 years old)	1	1
Middle (6–8 years old)	0.8077	0.84
Elder (10–14 years old)	1	1
